# The complete mitochondrial genome of *Mystus gulio* Hamilton (Siluriformes: Bagridae) and its phylogenetic implication

**DOI:** 10.1080/23802359.2023.2192311

**Published:** 2023-03-25

**Authors:** Hoang Danh Nguyen, Minh Thiet Vu, Hoang Dang Khoa Do

**Affiliations:** NTT Hi-Tech Institute, Nguyen Tat Thanh University, Ho Chi Minh City, Vietnam

**Keywords:** Bagridae, comparative genomics, long whiskers catfish, MinION

## Abstract

*Mystus gulio* Hamilton, also called long whiskers catfish, is an endemic fish and a common food in some Asian countries. In this study, the complete mitochondrial genome of *M. gulio* was sequenced using the MinION system (Oxford Nanopore Technologies). The mitochondrial genome is 16,518 bp in length (41.1% [G + C] content) and consists of 13 protein-coding genes, 22 transfer RNA genes, and two ribosomal RNA genes. The results of phylogenetic analysis inferred from whole mitochondrial genomes of *Mystus* and related species in the family Bagridae revealed that *M. gulio* is closely related to *Mystus cavasius*.

## Introduction

*Mystus gulio* Hamilton 1822, which belongs to the genus *Mystus* of the family Bagridae, order Siluriformes, is a small fish distributed in coastal waters from India to Indonesia and Vietnam (Talwar and Jhingran, 1991). It was assessed as a vulnerable species, and its population size is decreasing (Hossain et al. 2015). This fish, also known as “*cá chốt*” in Vietnamese, “*nuna tengra*” in Bengali, and “*pla kayeng noo*” in Thai (Froese and Pauly, 2022), has long been used in traditional food and has high commercial value in coastal fisheries of India, Bangladesh, Thailand, and Vietnam (Rahman et al. 2020). *M. gulio* is classified as a species of least concern in the International Union for Conservation of Nature Red List of Threatened Species; however, it is facing destructive fishing pressure, decreasing populations, habitat loss, and threats due to climate change (Hossain et al. 2015; Ng et al. 2019; Rahman et al. 2020). Although studies of the morphology, phylogeny, and reproductive biology of *M. gulio* have been conducted, there have been no extensive genomic studies (i.e. complete mitochondrial genome/mitogenome) of *M. gulio* (Gupta 2014; Kumar et al. 2019; Liu et al. 2019; Widayanti et al. 2021; Hashimoto et al. 2022). Therefore, we characterized the complete mitochondrial genome of *M. gulio* and analyzed its phylogenetic relationship within the family Bagridae. The new genomic data of *M. gulio* will provide fundamental information for further studies on the population genetics, evolution, and molecular markers of this species.

## Materials and methods

**Figure 1.**
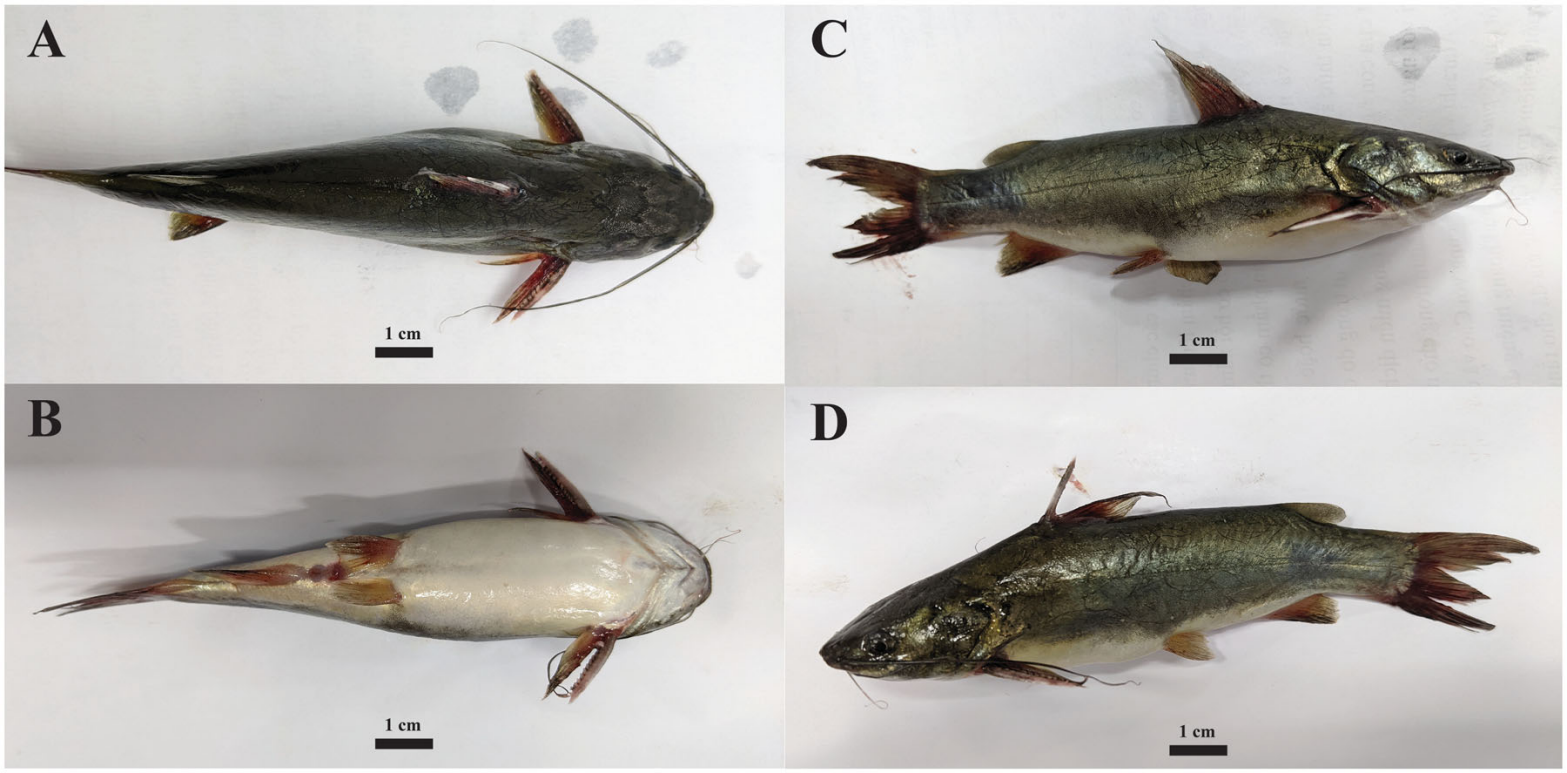
Morphology of *M. gulio*. A. the dorsal view. B. the ventral view. C. the left lateral view. D. the right lateral view. The photos were taken by the authors from Cantho city, Vietnam, on June 06^th^, 2022.

Samples (whole dead organisms) of *M. gulio* were collected in the Mekong Delta region, Can Tho City, Vietnam (10° 0′ 22.691″ N, 105° 45′ 3.311″ E). No specific permission was required to collect this species in Vietnam. The samples were stored at −80 °C and deposited at NTT Hi-Tech Institute – Nguyen Tat Thanh University (determined by Dr. Do Hoang Dang Khoa, email: dhdkhoa@ntt.edu.vn) under voucher number NTTU-202210-A001 (Figure 1). Total genomic DNA was extracted from liver tissue by the salt-out method (El-Ashram et al. 2016). To isolate the mitochondrial genome sequence, a long-range PCR approach was conducted using LongAmp^®^ Taq DNA Polymerase (#M0323S; New England Biolabs). The complete mitochondrial genome sequence of *Mystus cavasius* Hamilton 1822 (GenBank accession number NC_030187), *Mystus rhegma* Fowler 1935 (GenBank accession number NC_023223), and *Mystus vittatus* Bloch 1794 (GenBank accession number NC_032082) were used to design two primer pairs using Primer3 (Untergasser et al. 2012): 16SF (5′-CGCCTGTTTATCAAAAACAT-3′)/CytobR (5′-GGAATGCGAAGAATCGTGTT-3′) and trnLF (5′-CTCTTGGTGCAANTCCAAG-3′)/16SR (5′-CCGGTCTGAACTCAGATCACGT-3′), which amplified PCR products of 13 kb and 7 kb, respectively. The PCR protocol consisted of 2 min at 94 °C, followed by 35 cycles of 10 s at 94 °C, 30 s at 50 °C, and 6 min at 65 °C, with an extension step of 2 min at 65 °C. The PCR products were checked by 1% agarose gel electrophoresis. The amplicons were pooled before purification using the Monarch PCR & DNA cleanup kit (#T1030, New England Biolabs). The purified DNA was then used to prepare a sequencing library using Native Barcoding Expansion 1-12 (#EXP-NBD104, Oxford Nanopore Technologies) and the Ligation Sequencing Kit (SQK-LSK109, Oxford Nanopore Technologies) according to the manufacturer’s instructions. The prepared library was loaded into the MinION device with R9.4.1 flow cells (#FLO-MIN106, Oxford Nanopore Technologies) for sequencing. MinKNOW was used to monitor the sequencing process. Raw data (in fast5 format) were converted to fastq format using Guppybasecaller v5.0.7 (Wick et al. 2019). The fastq data were then assembled and the mitochondrial genome sequence of *M. gulio* was analyzed using Geneious Prime v2022.1 (https://www.geneious.com) with the mitochondrial genome of *M. cavasius* as a reference. The complete mitochondrial genome (average coverage depth = 257x) of *M. gulio* was annotated using GeSeq (Tillich et al. 2017). The complete mitochondrial sequence was deposited in GenBank under accession number OP650114. For phylogenetic analysis, the complete mitochondrial genomes of 45 species belonging to the Bagridae family and one species of Gymnotidae (*Gymnotus sylvius* Albert and Fernandes-Matioli 1999) was included as an outgroup. The sequences were aligned using MUSCLE v5 (Edgar 2004). The aligned data were tested for the best substitution model using jModelTest 2 (Darriba et al. 2012). The results showed that GTR + I + G is the best model for the data matrix of *Mystus* and related species. The maximum likelihood (ML) method was used for phylogenetic analysis with 1000 bootstrap replicates using IQ-TREE2 (Minh et al. 2020). For Bayesian inference (BI) analysis, the data matrix was analyzed using two Markov chain Monte Carlo runs for one million generations in MrBayes v3.2.7 (Huelsenbeck and Ronquist 2001). The tree was sampled every 1000 generations and 25% were discarded as burn-in. BI analysis was performed when the split frequency was lower than 0.01. The phylogenetic tree was modified and illustrated using Figtree v1.4.4 (http://tree.bio.ed.ac.uk/software/figtree/).

## Results

**Figure 2.**
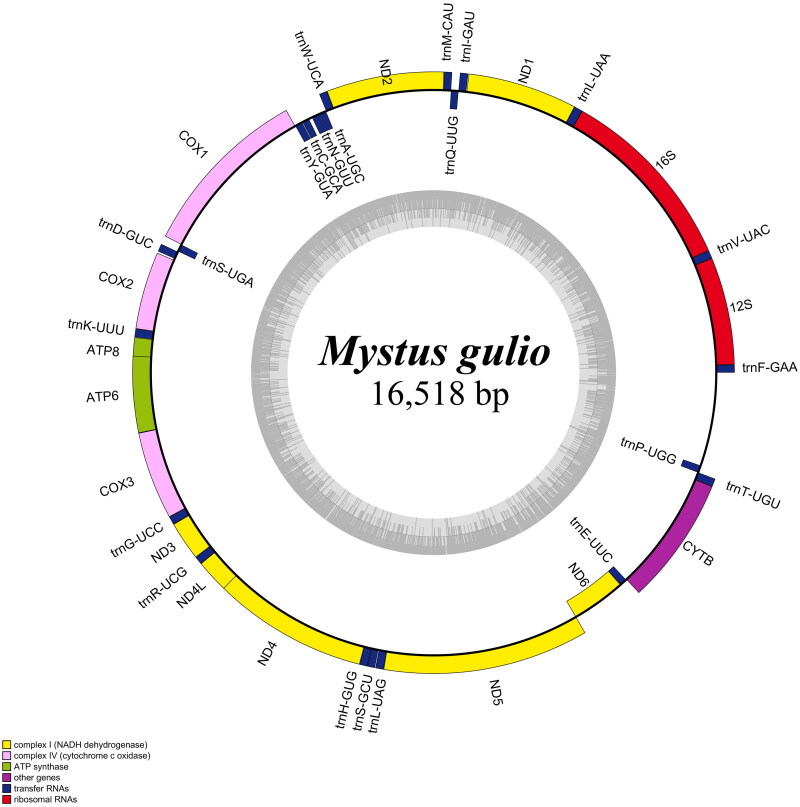
Map of the mitochondrial genome of *M. gulio*. The genes transcribed counterclockwise are located outside the circle, while genes transcribed clockwise are inside the circle. The dark gray area in the inner circle indicates the GC content of the mitochondrial genome, whereas the AT content is represented by a light gray area. Genes were classified into different functional groups that were shown in different color blocks.

The mitogenome of *M. gulio* was 16,518 bp in length (GenBank accession number OP650114) and included 22 transfer RNA (tRNAs) genes (*tRNAAla, tRNAArg, tRNAAsn, tRNAAsp, tRNACys, tRNAGln, tRNAGlu, tRNAGly, tRNAHis, tRNAIle, tRNALeu* (2×), *tRNALys, tRNAMet, tRNASer* (2×), *tRNAPhe, tRNAPro, tRNAThr, tRNATrp, tRNATyr*, and *tRNAVal*), 13 protein-coding genes (*ND1*, *ND2*, *COX1*, *COX2*, *ATP8*, *ATP6*, *COX3*, *ND3*, *ND4L*, *ND4*, *ND5*, *ND6*, and *CYTB*), and two rRNAs (12S rRNA and 16S rRNA; Figure 2). Among the protein-coding genes, *ND2, COX2, COX3, ND3, ND4,* and *CYTB* might have the fulfillment of incomplete stop codon T by posttranscriptional polyadenylation with poly-A tail (Ojala et al. 1981). The percentages of A, C, T, and G in the mitogenome were 31.9% (5265 nucleotides), 26.1% (4308 nucleotides), 27.0% (4459 nucleotides), and 15.1% (2486 nucleotides), respectively, with an [A + T] content of 58.9%. The gene content and order of the new mitogenome were similar to those of the published mitogenomes of other *Mystus* species. Specifically, the pairwise identities of mitogenomes of *M. gulio* compared with *M. cavasius*, *M. Rhegma*, and *M. vittatus* were 89.2%, 87.5%, and 81.6%, respectively.

**Figure 3.**
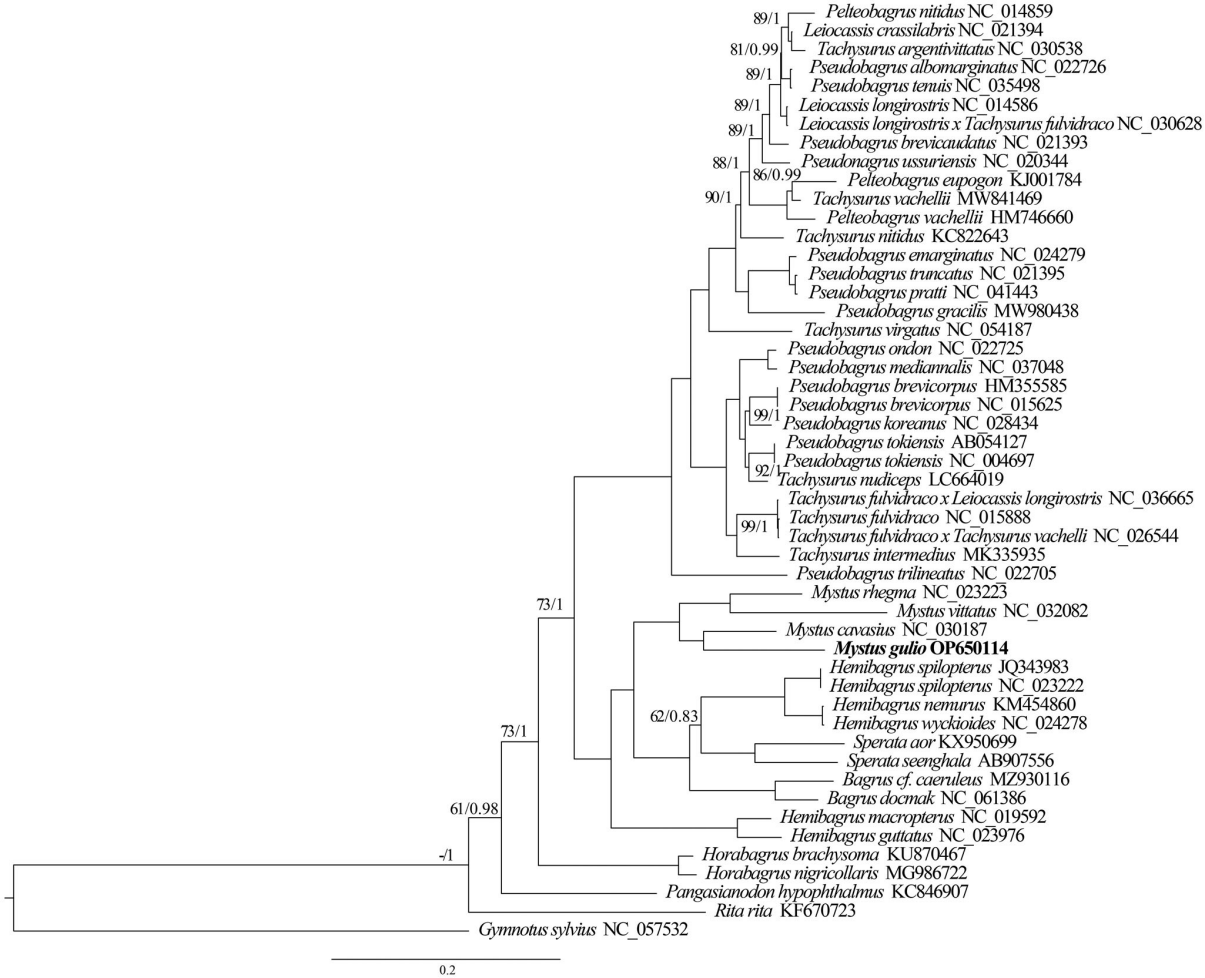
Phylogenetic tree of *Mystus* and related species in family Bagridae inferred from 46 whole mitochondrial genomes. The numbers indicate the bootstrap values (BS) and posterior probabilities (PP). Only the numbers (BS < 100 and PP < 1) were shown on the tree.

Phylogenetic analysis inferred from 46 complete mitogenomes revealed the same topology between the ML and BI methods (Figure 3). The monophyly of *Mystus* species was supported by high support values (bootstrap (BS)=100/posterior probability (PP)=1). In contrast, the paraphyly and polyphyly of *Hemibagrus*, *Pseudobagrus*, *Leiocassis*, and *Tachysurus* were found. Both BI and ML analyses supported a close relationship between *M. gulio* and *M. cavasius*.

## Discussion and conclusion

In this study, the complete mitochondrial genome of *M. gulio* was sequenced and characterized. The newly reported mitochondrial genome is similar in gene content and order to those of other *Mystus* species (Duan et al. 2019). In addition, the new genomic data revealed phylogenetic relationships between *M. gulio* and related species in Bagridae. Specifically, *M. gulio* is closely related to *M. Casavius*, which was reported in previous studies. The newly reported mitochondrial genome represents essential information for further studies on the evolutionary history, phylogeography, and conservation of *M. gulio*.

## Data Availability

The data generated in this study are openly available in the GenBank database https://www.ncbi.nlm.nih.gov/genbank/under the accession number OP650114. The associated BioProject, SRA, and BioSample numbers are: PRJNA889671, SRR21869731, and SAMN31249233 respectively
